# Single-beam digital holographic reconstruction: a phase-support enhanced complex wavefront on phase-only function for twin-image elimination

**DOI:** 10.1117/1.JBO.29.7.076502

**Published:** 2024-07-13

**Authors:** Charlotte Kyeremah, Matthew Weiss, Dila Kandel, Daniel Haehn, Chandra Yelleswarapu

**Affiliations:** a University of Massachusetts Boston, Department of Physics, Boston, Massachusetts, United States; b University of Massachusetts Boston, Department of Computer Science, Boston, Massachusetts, United States

**Keywords:** lensless in-line digital holographic microscopy, phase-only function, twin-image elimination, phase reconstruction, complex wave filtering, phase-support constraint

## Abstract

**Significance:**

In in-line digital holographic microscopy (DHM), twin-image artifacts pose a significant challenge, and reduction or complete elimination is essential for object reconstruction.

**Aim:**

To facilitate object reconstruction using a single hologram, significantly reduce inaccuracies, and avoid iterative processing, a digital holographic reconstruction algorithm called phase-support constraint on phase-only function (PCOF) is presented.

**Approach:**

In-line DHM simulations and tabletop experiments employing the sliding-window approach are used to compute the arithmetic mean and variance of the phase values in the reconstructed image. A support constraint mask, through variance thresholding, effectively enabled twin-image artifacts.

**Results:**

Quantitative evaluations using metrics such as mean squared error, peak signal-to-noise ratio, and mean structural similarity index show PCOF’s superior capability in eliminating twin-image artifacts and achieving high-fidelity reconstructions compared with conventional methods such as angular spectrum and iterative phase retrieval methods.

**Conclusions:**

PCOF stands as a promising approach to in-line digital holographic reconstruction, offering a robust solution to mitigate twin-image artifacts and enhance the fidelity of reconstructed objects.

## 1 Introduction

Digital holographic microscopy (DHM) is a powerful technique that enables capturing and reconstructing three-dimensional (3D) information of phase objects. By leveraging interference patterns created between objects and reference waves, information-rich reconstructed images with high axial and diffraction-limited transverse resolutions are acquired.^1^^–^^9^ Various configurations such as Gabor’s in-line^1^^,^^10^^,^^11^ and Leith-Upatnieks’ off-axis^12^^,^^13^ have been explored to reconstruct the object information from the recorded hologram, each offering its own set of advantages and disadvantages.

In off-axis DHM, the object and reference beams travel separate paths and interfere at the image plane with some angle between them. Even though this scheme eases the reconstruction process, it comes with trade-offs, specifically requiring vibration isolation and underutilization of the recording medium’s space-bandwidth product (SBP). By contrast, in the in-line DHM setup, both object and reference wavefields travel along the same path, allowing for full utilization of the SBP without the need for vibration isolation. However, this setup introduces undesirable twin-image artifacts due to the coincidence of virtual and real images, resulting in an apparent superimposition of out-of-focus images when focusing on either of them, which poses a significant challenge during object reconstruction.

Several techniques have been developed to reduce or eliminate twin-image artifacts. These include conventional diffraction methods such as the Fresnel or the angular spectrum method (ASM),^14^^–^^19^ linear filtering,^20^^,^^21^ iterative phase retrieval (ItPR) methods,^22^^–^^28^ deep learning,^29^^–^^32^ sparsity-based phase retrieval,^33^^–^^35^ and complex wavefront filtering.^25^^,^^36^^–^^38^ ASM involves computing wave propagation through Fourier transforms, enabling analysis of diffraction patterns and reconstructing wavefronts at various distances. It is a widely used numerical approach; however, it cannot effectively eliminate/reduce the unwanted twin-image artifacts, leading to a degradation in the reconstructed object’s quality. Linear filtering techniques alter the spatial or frequency components of the holographic data using filters to selectively remove undesirable components associated with twin images. It is quick and computationally efficient, especially for simple objects. However, it is effective only for simple objects,^38^ and it may introduce artifacts when applied indiscriminately.

Deep learning employs neural network architectures trained on holographic data to directly learn the mapping between holograms and their corresponding object waves.^29^^–^^32^^,^^39^^,^^40^ This approach effectively mitigates twin-image artifacts but requires a large dataset for training. In addition, complex neural networks may be computationally intensive and require substantial computational resources. By contrast, the sparsity-based method exploits the inherent sparsity within the object domain to improve holographic reconstruction.^33^^–^^35^ It can provide high-quality reconstructions with reduced artifacts. However, its performance may vary depending on the sparsity level of the object.

ItPR methods iteratively refine an estimate of the phase until a satisfactory reconstruction is achieved, thus involving an intensive iterative computational process and time. In addition, convergence may not always be guaranteed, and the choice of initialization parameters can significantly impact performance. Complex wavefront filtering techniques manipulate the complex-valued holographic data directly to suppress twin-image artifacts. They can provide high-quality reconstructions without extensive computational overhead. However, designing effective filters may require a deep understanding of the underlying physics and may not always be straightforward for complex scenes.

It is well known that phase is an essential aspect of an image as it contains key features of the object.^41^ Phase manipulation has been exploited to process images and extract the features of interest.^42^^–^^45^ As the phase-only information of the object contains all the required information, here, we report a novel approach called phase-support constraint on phase-only function (PCOF), wherein complex wave filtering is applied to the phase-only function. This technique facilitates object reconstruction using a single hologram, significantly reducing inaccuracies and avoiding iterative processing. Through in-line DHM simulations and tabletop experimental setups involving microbeads, non-centrosymmetric simulated objects, and biological specimens, PCOF’s effectiveness in eliminating the twin-image phenomenon is demonstrated. Quantitative assessments employing metrics such as mean squared error (MSE), peak signal-to-noise ratio (PSNR), and mean structural similarity index (MSSIM) underscore PCOF’s capacity for high-fidelity reconstruction, minimizing deviation from the original image. Furthermore, our method established superior performance on these metrics compared with conventional ASM and ItPR methods. This contribution holds promise for advancing holographic reconstruction methodologies, mitigating artifact-related challenges, and ensuring precise object representation.

## 2 Methodology

**Fig. 1:**
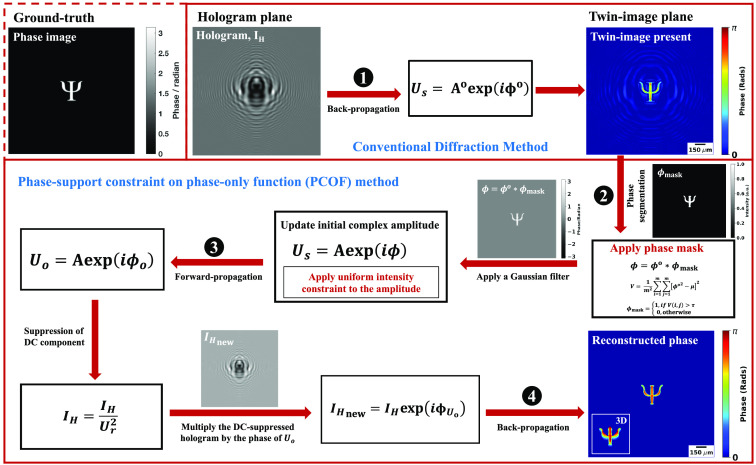
Workflow of the proposed reconstruction algorithm for twin-image elimination. A computer-generated hologram of a simulated phase object, Ψ with a phase step, π is used as the test object. Step 1 depicts the reconstruction using a conventional diffraction method, also known as the ASM. Steps 2 to 4 illustrate the PCOF, clearly demonstrating the suppression of the twin image.

Figure 1 presents a computer-generated hologram depicting a symbol Ψ as the simulated phase object with a phase step π, along with the algorithmic steps for its reconstruction. Utilizing a sliding-window technique, different parts of the reconstructed image were systematically analyzed by considering subsets or patches of pixels at a time. At each position, the arithmetic means and variance of pixel values within the window were computed, enabling the distinction of areas of significance solely based on phase values. Then, a support constraint mask was formulated through variance thresholding to effectively mitigate twin-image artifacts.

**Step 1: Initialization and back-propagation**

The first step in this proposed method is preprocessing the recorded hologram for contrast and image enhancements. The preprocessed holographic data are then initialized by creating an initial phase of zeros, and the ASM is employed to simulate wave propagation from the hologram plane to the object plane and vice versa. The wavefront $U_{s}(x,y,z)$ at a distance z from the hologram plane is computed using Eq. (1):

$$U_{s}(x,y,z)=F^{-1}\{F\{I_{H}(x^{\prime },y^{\prime },0)\}\cdot H_{BP}(f_{x},f_{y})\}\Leftrightarrow A^{o}e^{i\phi^{o}(x,y)},$$ (1)

where $I_{H}(x^{\prime },y^{\prime },0)$ is the preprocessed holographic field and F and $F^{-1}$ are the Fourier and inverse Fourier transforms, respectively. The initial phase, $\phi^{o}(x,y)$ obtained from the reconstructed hologram which has the twin image $\left(A^{o}e^{i\phi^{o}(x,y)}\right)$ present, is given by

$$\phi^{o}(x,y)=\tan^{-1}[\frac{\text{Im}(U_{s}(x,y))}{\text{Re}(U_{s}(x,y))}].$$ (2)

**Step 2: Segmentation of the twin image**

A segmentation mask is created through thresholding of a variance map derived from the reconstructed phase, $\phi^{o}(x,y)$ given by Eq. (2).

### 2.1 Calculation of the Variance Map

A variance map, V(x,y), is calculated from the in-focus reconstructed twin image based on the difference between the squared phase values and the arithmetic mean of local pixel values. This variance is a measure of local phase changes and indicates regions with significant features or abrupt phase changes. A specified window size of 5×5 is chosen, and the phase of the reconstructed twin image is processed by calculating the variance in the overlapping blocks using Eq. (3):

$$V=\frac{1}{m^{2}}\overset{m}{\underset{i=1}{\sum }}\overset{m}{\underset{j=1}{\sum }}[\phi^{o}{\left(x,y\right)}^{2}-\mu {]}^{2},$$ (3)

where $\mu =(\frac{1}{m^{2}}\sum_{i=1}^{m}\sum_{j=1}^{m}[\phi^{o}(x,y)])$ is the arithmetic mean of the current block m×m pixels.

### 2.2 Thresholding and Masking

A mask that identifies regions of interest within the holographic reconstruction is created. This mask is derived from a threshold applied to the variance map. A threshold, τ, is set based on a percentage of the maximum phase value [using Eq. (2), 50% of the maximum phase value is used here], and pixels with a variance above this threshold are considered part of the segmented region. The threshold value can be adjusted based on the propagation distance, z. A binary mask,

$$\phi_{\text{mask}}=\{\begin{array}{ll} 1, & \text{if }V(i,j)>\tau \\ 0, & \text{otherwise} \end{array},$$ (4)

is then created based on the thresholding, where pixels above the threshold are set to 1, while the segmented region and pixels below the threshold are set to 0. This enabled the removal/elimination of the unwanted region (twin-image artifacts). The phase mask, $\phi_{\text{mask}}$, is then used to create a new phase (propagated phase mask) using Eq. (5), effectively focusing the reconstruction on regions with high variance. It acts as a constraint, allowing areas with significant phase variations to be emphasized,

$$\phi (x,y)=\phi^{o}(x,y)\cdot \;\phi_{\text{mask}}.$$ (5)

A Gaussian blur filter is then applied to the updated phase, ϕ(x,y) to reduce noise and smoothen the phase information.

**Step 3: Object constraint and forward-propagation**

The phase-only function is calculated to preserve key features of the object in the reconstructed image. This approach involves selecting only the phase of, $U_{s}(x,y)=Ae^{i\phi (x,y)}$, while making the amplitude unity. This process eliminates amplitude with negative values as the intensity cannot be negative. Then, the transmission function is updated, and forward propagation is performed to obtain a new holographic field where the amplitude is replaced by the recorded hologram

$$U_{o}(x,y)=F^{-1}\{F\{(U_{s}(x,y))\}\cdot \;H_{FP}(f_{x},f_{y})\}\Leftrightarrow Ae^{i\phi_{o}(x,y)}.$$ (6)

The recorded hologram, $I_{H}(x^{\prime },y^{\prime },0)$, is filtered by dividing the hologram by the square of the reference beam, $U_{r}(x,y)$ using Eq. (7) to suppress the direct current component and create a new hologram

$$I_{H}(x,y)=\frac{I_{H}(x^{\prime },y^{\prime })}{U_{r}^{2}(x,y)}.$$ (7)

**Step 4: Object reconstruction and back-propagation**

The new hologram free from the twin-image artifacts is obtained by multiplying the filtered hologram, $I_{H}(x,y)$ with the complex exponential of the phase angle of $U_{o}(x,y)$

$$I_{H_{\text{new}}}(x,y)=I_{H}(x,y)\times e^{i\phi_{U_{o}}(x,y)}.$$ (8)

This operation combines the amplitude of the filtered hologram with the phase-only information of the output, $U_{o}(x,y)$ [Eq. (6)]. The final backpropagation is performed to obtain the reconstructed holographic field for further analysis to extract the true phase (unwrapped phase) map of the object

$$U_{obj}(x,y)=F^{-1}\{F\{I_{H_{\text{new}}}(x,y)\}\cdot H_{BP}(f_{x},f_{y})\}\Leftrightarrow Ae^{i\phi_{obj}(x,y)}.$$ (9)

### 2.3 Evaluation of the Algorithm and Image Quality

To facilitate a comprehensive assessment of various algorithms and their corresponding reconstruction outcomes, MSE is used as a metric to quantify the error or discrepancy between the ground truth object and its reconstructed image. The MSE is normally defined as follows:

$$\mathrm{MSE}(i,j)=\frac{1}{M\times N}\overset{M}{\underset{i=1\cdots M}{\sum }}\overset{N}{\underset{j=1\cdots N}{\sum }}{\left[\rho -\rho_{o}\right]}^{2},$$ (10)

where ρ(i,j) is the reconstructed distribution, $\rho_{o}(i,j)$ is the initial distribution, and [N, M] is the number of pixels. The MSE, calculated as the average of squared differences among pixel values, serves as an indicator of how closely the pixel values in the reconstructed image resemble those in the original image. A lower MSE signifies a closer match between two images.

PSNR is another metric used to measure the quality of reconstructed images compared with the original images. It is a logarithmic ratio of the maximum possible pixel value to the square root of the MSE.

$$\mathrm{PSNR}=10\cdot \log_{10}{\left(\frac{\max(\rho )}{\mathrm{MSE}}\right)}^{2},$$ (11)

where max(ρ) is the maximum possible pixel value of the object’s phase map. Since PSNR is inversely related to the MSE, as the MSE decreases, the PSNR increases, indicating a higher-quality reconstruction. A higher PSNR denotes increased similarity between the original and reconstructed images.

MSSIM is a metric used to quantify the similarity between two images $\left(\rho ,\rho_{o}\right)$. It assesses structural information preservation during image processing. The MSSIM value ranges between −1 and 1, where 1 indicates perfect similarity, 0 denotes no similarity, and negative values suggest dissimilarity. A higher MSSIM value implies better preservation of structural details between the original and reconstructed images.

$$\mathrm{SSIM}(\rho ,\rho_{o})=\frac{\left[2\mu_{\rho }\mu_{\rho_{O}}+C_{1}]\times [2\sigma_{\rho \rho_{O}}+C_{2}\right]}{\left[\mu_{\rho }^{2}+\mu_{\rho_{O}}^{2}+C_{1}]\times [\sigma_{\rho }^{2}+\sigma_{\rho_{O}}^{2}+C_{2}\right]},$$ (12)

where $\mu_{\rho }$, $\mu_{\rho_{O}}$, $\sigma_{\rho }$, $\sigma_{\rho_{O}}$, and $\sigma_{\rho \rho_{O}}$ are the average luminance of images, standard deviations of the pixel intensities, and the covariance of the pixel intensities in images ρ and $\rho_{o}$. $C_{1}$ and $C_{2}$ are constants added for numerical stability. MSSIM is computed by comparing local patterns of pixel intensities in both images, offering insights into structural similarity. A higher MSSIM signifies a greater resemblance between the structures of the original and reconstructed images.

## 3 Results and Discussion

**Fig. 2:**
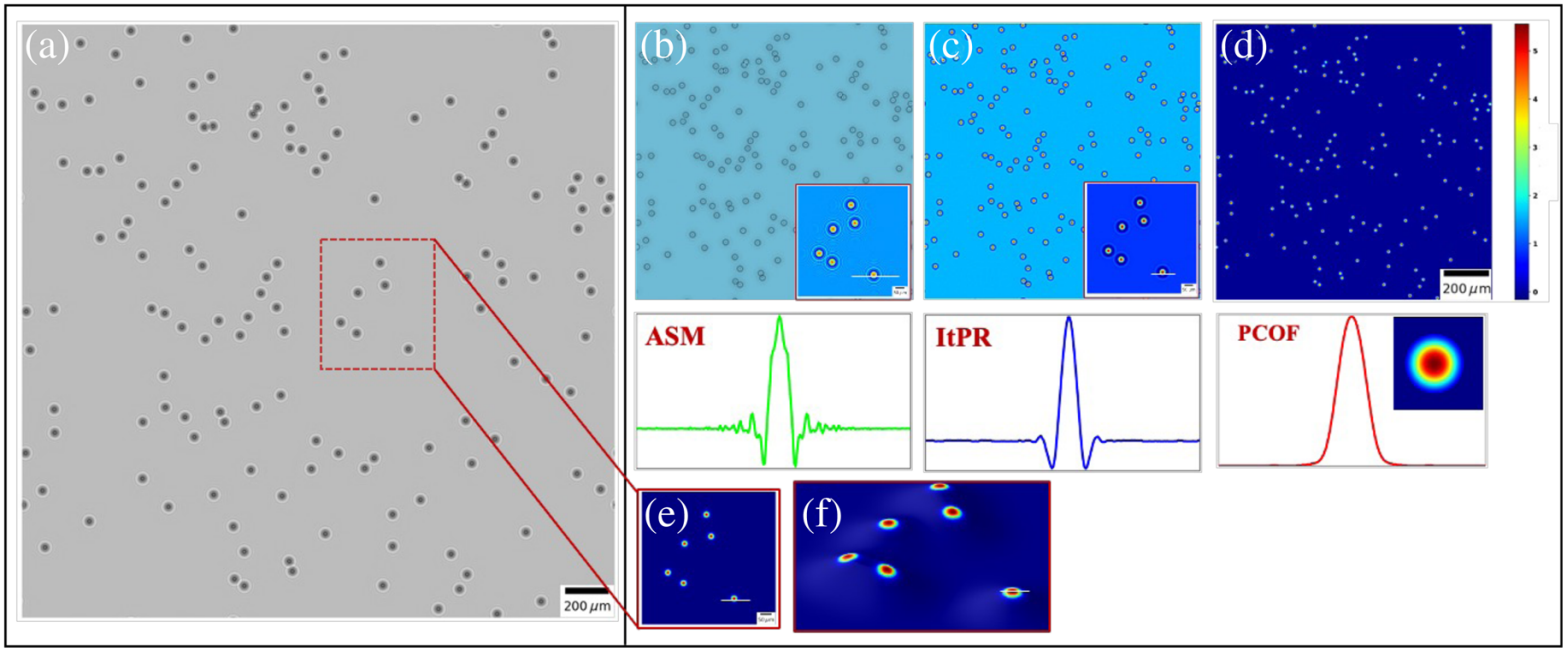
Reconstruction and comparison of simulated 30  μm beads. (a) Full FOV hologram of 150 beads; panels (b)–(d) are the reconstructed phase of the region of interest (ROI) (red box) by ASM, ItPR after 30 iterations, and PCOF methods. The inset is the phase profile of the selected single bead; panels (e) and (f) are the 2D and 3D profiles of the ROI by PCOF, respectively. The 3D profile shows that the PCOF can be used to reconstruct objects at different depths. The phase profiles of panels (b)–(d) are for observation.

The efficacy of our proposed methodology is demonstrated by recording and reconstructing microscopic objects via both in-line DHM simulations and tabletop experimental setups. Ground truth objects were generated with precise optical properties for the simulation phase. For instance, a configuration of 150 microsphere beads, each measuring a diameter of 30  μm and a phase step of 2π, was simulated [depicted in Fig. 2(a)]. Holograms were generated using the angular spectrum propagation function and then reconstructed using the proposed approach. The reconstructions were compared against established ASM^14^^,^^17^^,^^19^ and ItPR^26^^,^^46^ methods. Figures 2(b)–2(d) show the reconstructed microspheres using ASM, ItPR, and the PCOF methods, respectively. The phase profiles of the reconstructed images show “ringing” around reconstructed phase object information in the case of ASM and ItPR, whereas no ringing is observed in the PCOF reconstruction. The absence of ringing highlights its superior performance in effectively suppressing twin-image artifacts and ensures accurate representation and true preservation of the object information. The maximum phase value obtained post-reconstruction closely matched the predefined maximum phase shift of 2π radians, differing by only 0.312 radians.

To provide a deeper understanding of fidelity, quality, and similarity between the original and reconstructed images, quantitative analysis using multiple metrics, such as the MSE, PSNR, and MSSIM, was performed, and the values are presented in Table 1. Both ASM and ItPR have an MSE of 0.02, indicating a greater deviation from the original image. By contrast, PCOF exhibited an exceptionally low MSE of 0.01, signifying a closer match between the pixel values in the reconstructed and original images. The PSNR values of 65.56 dB for the ASM, 66.78 dB for the ItPR method, and 68.40 dB for PCOF reflect varying reconstruction qualities, with the proposed method outperforming both other methods. The MSSIM values further support these findings. The ItPR method achieves substantial structural similarity with an MSSIM of 0.9987 but falls short of near-perfect resemblance. By contrast, the ASM’s MSSIM of 0.9980 indicates relatively lower structural similarity compared with the other methods. Remarkably, PCOF achieves an almost perfect structural similarity, with an MSSIM of 0.9992, highlighting its exceptional ability to reconstruct images closely resembling the original data. Overall, the PCOF method outperforms ASM and ItPR methods, with lower MSE, higher PSNR, and markedly higher MSSIM values. These findings highlight PCOF’s exceptional capability for high-fidelity reconstructions, affirming its effectiveness in digital holographic image reconstruction.

**Table 1:** Quantitative metrics were obtained for the reconstruction of the simulated microbeads using the named reconstruction methods.

Method	MSE	PSNR (dB)	MSSIM
ASM	0.02	65.56	0.9980
ItPR	0.02	66.78	0.9987
PCOF	0.01	68.40	0.9992

**Fig. 3:**
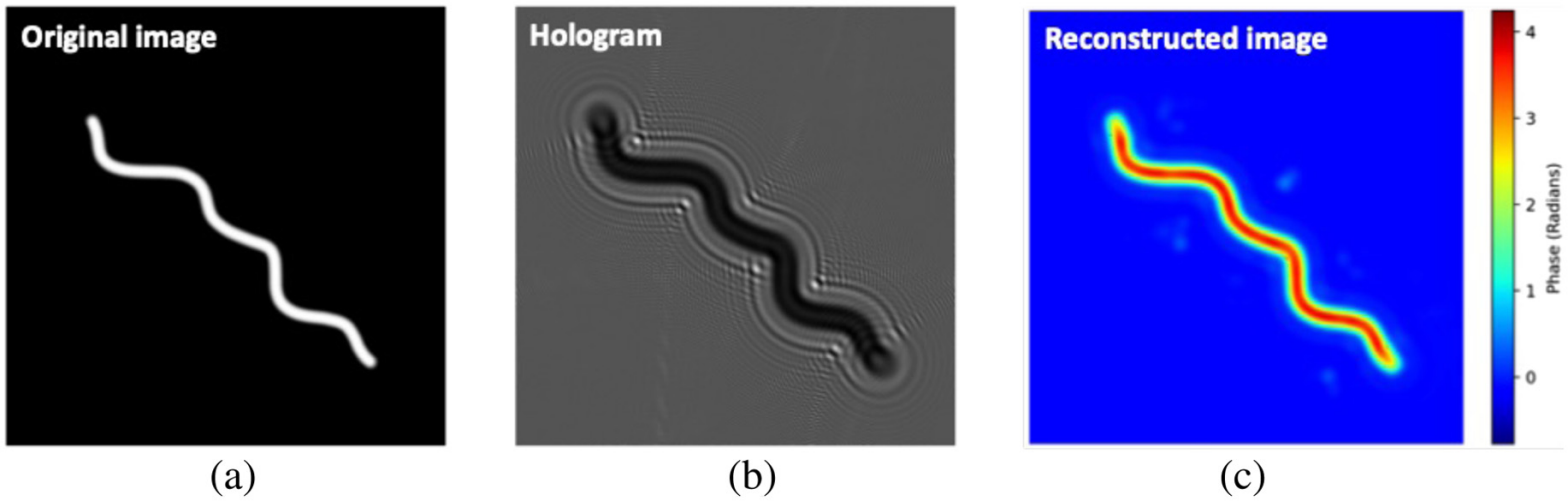
Simulated and reconstructed hologram of an extended object. (a) The ground truth image, (b) a recorded hologram, and (c) a reconstructed image of the extended object using the PCOF method.

To demonstrate the application of the PCOF for non-centrosymmetric objects, an extended object resembling a spiral was simulated.^46^ The reconstruction employed a threshold set at 50% of the maximum phase value. The resulting output, as shown in Fig. 3, indicates high fidelity and substantial structural similarity between the original and reconstructed images. The quantitative evaluation produced an MSE of 0.02, a PSNR of 64.99 dB, and an MSSIM of 0.9970.

### 3.1 Experimental Verification

A tabletop in-line lensless DHM system was built to study the effectiveness of PCOF in analyzing real-phase objects. The setup comprises a partially coherent light emitting diode (LED) light source (Thorlabs, LED525E, Newton, New Jersey, United States, 2.6 mW @ 20 mA, wavelength λ=525  nm), which passes through a 50  μm pinhole for spatial filtering. The sample is positioned ∼10  cm from the light source by employing detector plane holography, while the detector sits ∼1  mm away. This arrangement yields a field of view (FOV) equivalent to the active area of the detector, $∼30\;{\mathrm{mm}}^{2}$ for a Raspberry Pi camera boasting a pixel size of 1.55  μm and a resolution of 4056×3040   pixels.

**Fig. 4:**
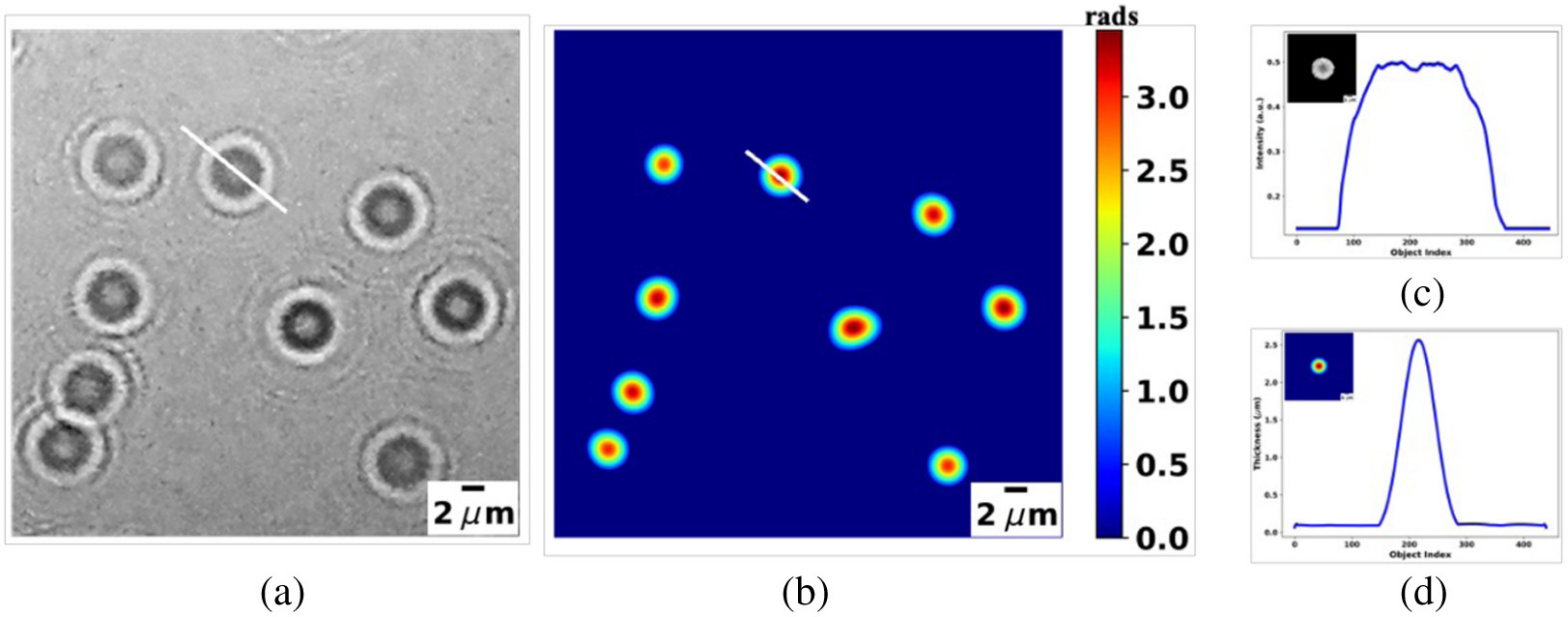
Phase characterization and thickness profile of silica beads. (a) Hologram of silica beads of 2.5  μm size, (b) reconstructed phase, (c) reconstructed intensity/amplitude of the white-lined single bead, and (d) the thickness profile of the reconstructed single bead.

The imaging of red blood cells over a large FOV and the subsequent generation of 3D phase maps offer a significant value for blood and disease analyses. Despite the relatively low range of phase values, the sample’s thickness can be accurately calculated following phase characterization using a known reference sample. To illustrate the utility of PCOF for such analysis, phase characterization through the imaging of 2.5  μm polystyrene silica beads (Polysciences, Inc., Warrington, Pennsylvania, United States) with a refractive index of 1.43 was conducted. The beads were suspended in water ($n_{\mathrm{med}}=1.33$) and sandwiched between two coverslips. The hologram of the beads was captured using the in-line lensless (DHM) setup. The phase reconstruction obtained using PCOF is depicted in Fig. 4(b). Analysis of the selected glass bead within the region of interest revealed an average size of 2.55±0.20  μm, aligning closely with the manufacturer’s specifications.

**Fig. 5:**
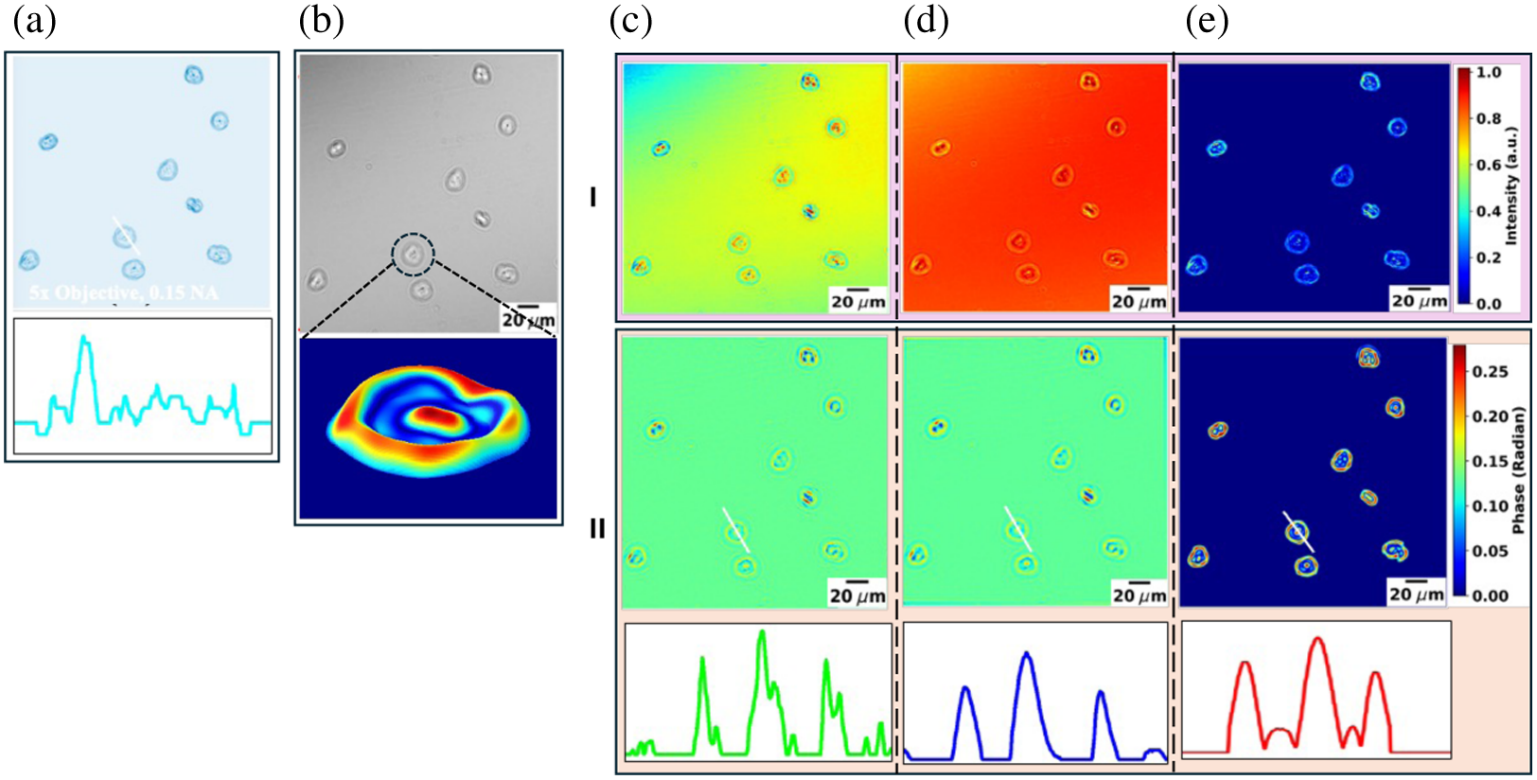
Hologram recording and reconstruction of cheek cells. (a) Brightfield image obtained using a 5×0.15 numerical aperture microscope objective, and the intensity profile; (b) in-line hologram and 3D phase maps obtained using PCOF; (c)–(e) reconstructions using ASM, ItPR, and PCOF methods, respectively; I: intensity reconstruction; II: phase reconstruction (top row) and phase profile (bottom row). Intensity and phase profiles of a cheek cell are obtained along the indicated white line.

Further imaging and reconstruction of the cheek cell sample are depicted in Fig. 5. Initially, the sample was verified using a standard bright-field microscope, as shown in Fig. 5(a). Subsequently, the sample was imaged using an in-line DHM setup, and the unwrapped phase was obtained, as shown in Fig. 5(b). Intensity (box I) and phase reconstruction (box II) were performed using the ASM [Fig. 5(c)], ItPR [Fig. 5(d)], and PCOF [Fig. 5(e)] methods. The bottom row of box II displays phase profiles. The results demonstrated that the PCOF method significantly reduced twin-image artifacts present in the ASM and ItPR reconstructions. Furthermore, the unwrapped image reveals all the internal structures of the cell, including the nucleus, cytoplasm, and cell membrane.

It can be observed that minimal residual speckle noise is present in our images. However, it is important to note that such noise is typical in coherent or partially coherent imaging. Although LEDs produce less speckle than lasers, the use of a pinhole in the in-line DHM setup can still introduce some speckle noise due to diffraction and scattering. The phase-support constraint in our method helps to limit the phase values to a defined support region, reducing the influence of noise outside this region. By focusing the reconstruction process within the defined support, the method effectively filtered out noise and artifacts that are not consistent with the expected phase distribution.

## 4 Conclusion

The significance of phase information in imaging techniques motivated the development of complex wavefront using PCOF as a novel approach to tackle twin-image artifacts in digital holography microscopy. PCOF’s broad utility was showcased through extensive simulations, tabletop experiments, and quantitative evaluations utilizing various metrics obtained with cheek cells, glass beads, and non-centrosymmetric objects. Comparative analysis with conventional methods such as ASM and ItPR highlighted PCOF’s superiority in terms of MSE, PSNR, and MSSIM values, emphasizing its potential for advancing holographic reconstruction methodologies. PCOF offers a promising solution by swiftly suppressing twin-image artifacts and accurately reconstructing the object phase without iterative processes, thereby ensuring high-quality holographic image reconstructions. Future endeavors could involve exploring the application of the developed system in real-time imaging and diagnostics, which holds promise for further investigation.

## Data Availability

Data underlying the results may be obtained from the authors upon reasonable request.
